# Prediction of postoperative mortality in elderly patient with hip fractures: a single-centre, retrospective cohort study

**DOI:** 10.1186/s12871-018-0646-x

**Published:** 2018-12-03

**Authors:** Romain Niessen, Benoit Bihin, Maximilien Gourdin, Jean-Cyr Yombi, Olivier Cornu, Patrice Forget

**Affiliations:** 1 Department of Anesthesiology, Université catholique de Louvain (UCL), CHU UCL Namur, 1 avenue Dr G Therasse, 5530 Yvoir, Namur, Belgium; 2 Scientific support unit, Université catholique de Louvain (UCL), CHU UCL Namur, 1 avenue Dr G Therasse, 5530 Yvoir, Namur, Belgium; 3 Department of Anesthesiology and scientific support unit, Université catholique de Louvain (UCL), CHU UCL Namur, 1 avenue Dr G Therasse, 5530 Yvoir, Namur, Belgium; 4 Department of Internal and Peri-operative Medicine, Université catholique de Louvain (UCL), Cliniques universitaires Saint-Luc, Brussels, Belgium; 5 Department of Orthopaedic surgery, Université catholique de Louvain (UCL), Cliniques universitaires Saint-Luc, Brussels, Belgium; 6 Department of Anesthesiology and Peri-operative Medicine, Vrije Universiteit Brussel (VUB), Universitair Ziekenhuis Brussel (UZ Brussel), Brussels, Belgium

**Keywords:** Biomarkers, C–reactive protein, Frailty, Hip fractures, Hospital mortality

## Abstract

**Background:**

Elderly patients are at high risk for postoperative complications and increased mortality after hip fracture (HF) surgery due to frailty and co-morbidities. The prediction of postoperative outcome could be used for clinical decision making. A reliable score to predict postoperative mortality after HF surgery in this sub-population remains unavailable.

**Methods:**

A single-centre retrospective cohort study was performed in 782 patients who were operated on for HF. Receiver Operating Characteristic (ROC)-curves were used to analyse the performance of gender, age, neutrophil-to-lymphocyte ratio (NLR) and C-reactive protein (CRP) at admission (D_0_) as prognostic factors, alone or combined with the PreOperative Score to predict PostOperative Mortality (POSPOM) in univariate and multivariate linear regression models.

**Results:**

No correlation between gender, age, NLR D_0_ or CRP D_0_ and postoperative, intra-hospital mortality was found. The Area Under the ROC-curve (AUC) for age, male gender, NLR and CRP were 0.61 [95% confidence interval (CI) = 0.45–0.61], 0.56 [95% CI = 0.42–0.56], 0.47 [95% CI = 0.29–0.47] and 0.49 [95% CI = 0.31–0.49] respectively. Combination with the POSPOM score did not increase its discriminative capacity as neither age (AUC = 0.69, 95% CI = 0.54–0.69), gender (AUC = 0.72, 95% CI = 0.58–0.72), NLR D_0_ (AUC = 0.71, 95% CI = 0.56–0.71), nor the CRP D_0_ (AUC = 0.71, 95% CI = 0.58–0.71) improved the POSPOM performance.

**Conclusions:**

Neither age, gender, NLR D_0_ nor CRP D_0_ are suitable parameters to predict postoperative, intra-hospital mortality in elderly patients undergoing surgery for HF.

## Background

Elderly patients are at high risk for postoperative complications and increased mortality after hip fracture (HF) surgery due to frailty and co-morbidities. According to available studies, one-year mortality after HF surgery ranges from 12 to 37% [1]. The prediction of postoperative outcome could help with clinical decision making. In previous years, several postoperative mortality scores have been developed for this specific purpose in the setting of elective surgeries. However, these scores often misinterpret orthogeriatric patients’ outcomes and risk of death [2–4].

The PICO model was used to clearly define a clinical question. The population is represented by elderly patients with hip fractures. The intervention aims to identify prognostic factors which improve the accuracy of postoperative mortality prediction in the specific sub-population. Four potential prognostic factors were examined, namely: CRP D_0_, NLR D_0_, age and gender. C-reactive protein (CRP) and the neutrophil-to-lymphocyte ratio (NLR) reflect the inflammatory status of patients during the peri-operative period. Furthermore, the NLR at admission (D_0)_ and at the fifth day (D_5_) have been shown to be associated with postoperative complications [3, 5–7]. We compared predicted mortality to observed mortality. The outcome of the study is to improve mortality prediction by analysing if the addition of these variables to the POSPOM score, one of the best validated prognostic tools in the peri-operative period, may increase its discriminative capacity.

## Methods

This study is presented according to the STROBE guidelines (www.strobe-statement.org).

### Study design

Retrospective analysis of a single-centre cohort.

### Settings

Included patients were admitted to the university hospital of St. Luc in Belgium (*Cliniques universitaires Saint-Luc*) between 2010 and 2016. Study cases were directly recruited after diagnosis of hip fracture in the emergency department or surgical ward. Date of admission, date of discharge, date of intra-hospital death, age, sex, co-morbidities, NLR D_0_, CRP D_0_ values of patients were recorded. Follow-up was terminated upon hospital discharge or intra-hospital death. Data registration and management were performed in agreement with Belgian law and the Helsinki declaration.

### Participants

After receiving ethical committee approval (Commission d’Ethique Biomédicale Hospitalo-Facultaire - CEBHF) of the Catholic University of Louvain (Chairperson: Prof J-M Maloteaux, n°2010/23DEC/406), the authors were granted a waiver for written informed consent due to the retrospective nature of the study and analysis of anonymised data. The database included a total of 782 patients with a diagnosis of HF. Patients lacking personal data concerning co-morbidities were excluded from the study. All patients included in the study were treated following the same early surgical care protocol (i.e. 81% operated within the first 24 h). This methodology consisted of obtaining medical clearance from the emergency department as soon after the diagnosis as possible. Patients were then wait-listed and commonly operated the same day. In cases of treatment with anti-vitamin K medication, coagulation was restored with vitamin K. Patients under anti-platelets treatment were operated on without additional delay, in agreement with the surgeon, under general anaesthesia. When possible, regional anaesthesia/analgesia was proposed and performed, including a fascia iliaca block (single shot). Particular attention was paid to haemodynamic control, with the use of invasive blood pressure monitoring and dynamic variables when indicated and applicable. Postoperative follow-up was performed by an inter-disciplinary medical team consisting of orthopaedic surgeons, anaesthetists, geriatricians, an internal medicine specialist dedicated to peri-operative medicine, and physiotherapists.

### Variables

In previous studies, advanced age and male gender were identified as risk factors in patients with HF [3, 5]. Thus, age and gender were chosen as variables with potential discriminative capacity.

Age and gender were registered during pre-operative evaluation. Data on NLR D_0_ and CRP D_0_ were taken from the first blood sample obtained from the patient at admission and before surgery. In our clinical practice, blood testing is only realised in presence of an anamnestic or clinical problem, in order to not delay surgery. All blood analyses were performed on venous blood samples and were processed in a blood analyser (Sysmex; TAO Medical Electronics, Kobe, Japan) for full blood count and differential count of leukocytes. The NLR value was obtained by calculating the ratio between registered neutrophils and lymphocytes counts. The CRP value was determined based on a serum sample by a turbidimetry process (UniCel® DxC 800; Beckman Coulter, Pasadena, California, USA) and is expressed in mg l^− 1^.

The POSPOM score of each patient was calculated as the sum of the points assigned to each item (age, co-morbidities and type of surgery). With regards to the first variable (age), older patients received higher points. The second variable (co-morbidities) consisted of the total number of points assigned to each of the 17 validated co-morbidities. The third variable (type of surgery) was identical for all patients (“orthopaedic trauma”) and therefore each patient received 14 points. Depending on the total number of points, a percentage predicted risk of in-hospital mortality was assigned to each patient [8].

### Data collection

Data collection (Date of admission, date of discharge, date of intra-hospital death, age, sex, co-morbidities, NLR D_0_, CRP D_0_ values) was performed using systematic, standardised and computerised medical charts issued by the institutional software (Medical Explorer v9, Saint-Luc university Hospital, Brussels, 2009).

Control of potential biases was performed using a prospective listing and a standardisation of the data collection process. The increased weight of the variable “age” was intentional.

The population was divided into two groups: patients discharged from the hospital and patients who died in hospital. Quantitative variables such as the NLR, CRP and age were analysed in the descriptive analysis.

### Statistical methods

Pearson’s correlation coefficient was computed to identify potential linear association between log(NLR) and log(CRP).

In order to determine the performance of each individual variable (original POSPOM setting, age, gender, NLR and CRP) in the prediction of postoperative mortality, we computed the AUC with DeLong confidence intervals. A logistic regression model was then used to combine POSPOM score with the four other variables to determine whether the addition of one of these variables could improve the predictive value of the POSPOM score.

All analyses were performed using R 3.3.2 (R Foundation for Statistical Computing, 2016, Vienna, Austria) and the ggplot2 and pROC packages.

## Results

### Participants

**Fig. 1:**
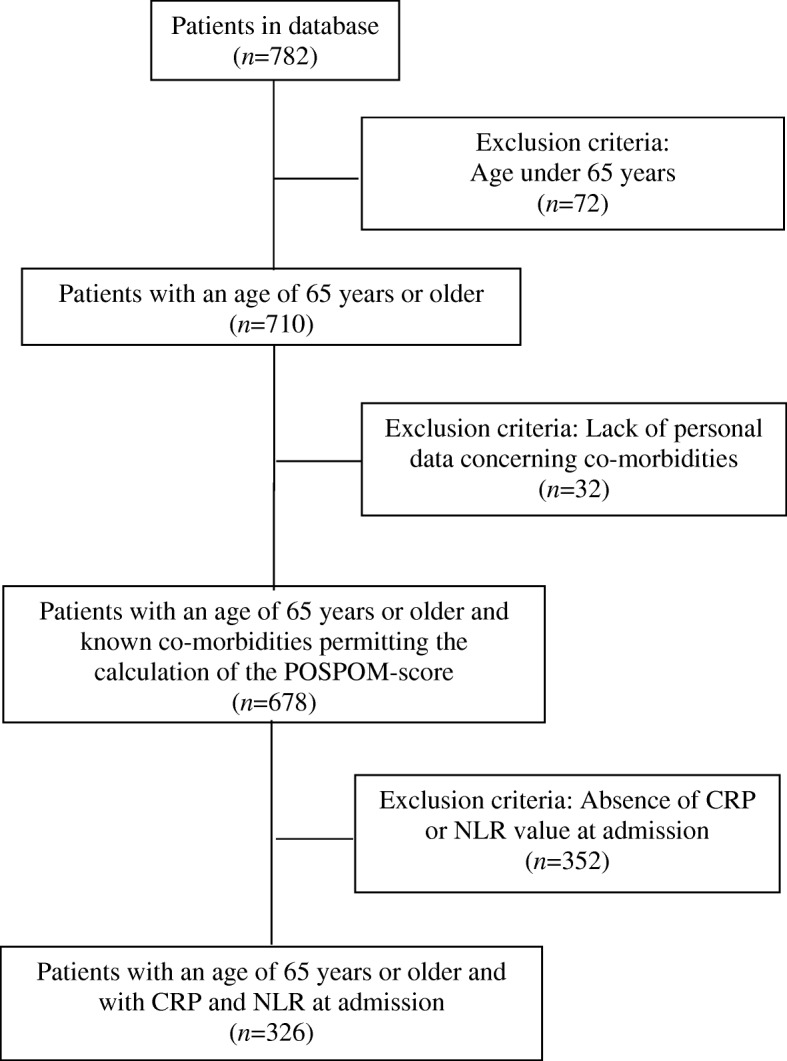
Patient flow chart diagram

Of the 782 included patients, 72 were aged under 65 years and 32 had a lack of personal data concerning co-morbidities. Of the 678 patients who were enrolled, 326 had NLR and CRP values at admission. Study flow chart including exclusion criteria is shown in Fig. 1.

### Descriptive and outcome data

The POSPOM-score predicted an average mortality of 13.24%, which is in contrast with the observed intra-hospital mortality of 4.5% (32 out of 678 patients). In women, the observed intra-hospital mortality was 3.9% (18 of 470 patients) and 6.3% in men (14 of 208 patients) (Table 1).

**Table 1:** Observed versus predicted mortality in female and male patients

Gender	Number of patients(*n*)	Observed Deaths (patients)	Observed Mortality (%)	Predicted mortality (%)
Female	470	18	3.6	11*.*61
Male	208	14	6.3	16*.*83
All	678	32	4.5	13*.*24

The mean age in surviving patients was 84 ± 7 years (minimum age = 65 years, maximum age = 105 years) versus 85 ± 9 years in non-survivors. The mean NLR D_0_ in survivors was 8 ± 6 and 10 ± 8 in non-survivors. The mean CRP D_0_ was 30.63 ± 52.71 mg l^− 1^ in survivors vs. 47.19 ± 73.83 in non-survivors. The mean POSPOM score was 30 in survivors and 32 in non-survivors, representing a predicted mortality of 13.24%. (Table 2).

**Table 2:** Descriptive analysis showing results of four quantitative variables

		Mean	SD	Median	Min	Max	NA
Age (years)	Survivors	83.649	7.390	85.0	65.0	105	0
	Non-survivors	85.312	9.146	87.5	65.0	100	0
	All	83.724	7.479	85.0	65.0	105	0
NLR D_0_ (ratio)	Survivors	8.125	5.826	6.4	0.5	45	136
	Non-survivors	9.393	5.236	7.2	2.1	27	7
	All	8.183	5.796	6.6	0.5	45	143
CRP D_0_ (mg/l)	Survivors	30.63	52.71	7.0	1.0	421.0	328
	Non-survivors	47.19	73.83	5.5	1.0	243.0	16
	All	31.35	53.78	7.0	1.0	421.0	344
POSPOM (score)	Survivors	30.063	5.397	30.0	9.0	44	0
	Non-survivors	31.594	4.918	32.0	14.0	41	0
	All	30.132	5.383	30.0	9.0	44	0

*SD* standard deviation, *NA* not applicable

### Main results

The calculated correlation coefficients between NLR and CRP showed weak correlation with a Pearson r of 0.216 [95% CI = 0.110–0.317] and a Spearman r of 0,230 [95% CI = 0.159–0.298].

**Fig. 2:**
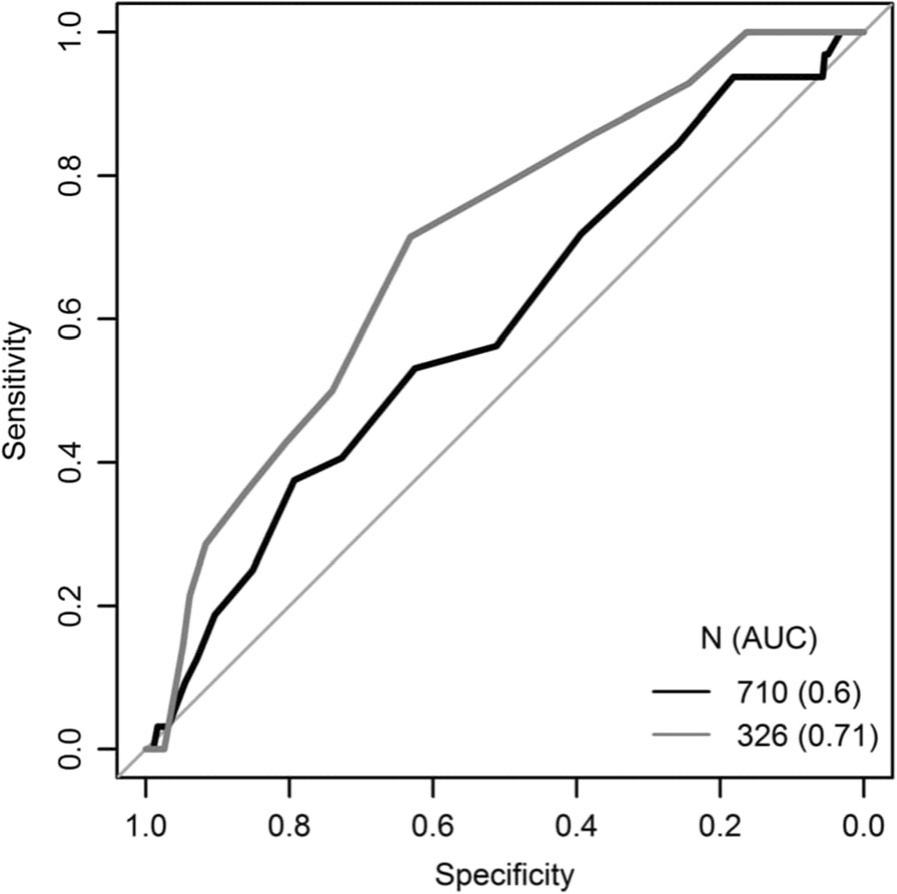
ROC curves showing complete dataset (after exclusion of patients with an age under 65 years) and restricted dataset of patients with NLR and CRP values. Legend: black line = complete dataset; grey line = restricted dataset

The AUC of the complete dataset showed a performance of 0.596 [95% CI = 0.494–0.596] versus an AUC of 0.705 [95% CI = 0.572–0.705] in the restricted dataset with an available NLR and CRP. (Fig. 2).

**Fig. 3:**
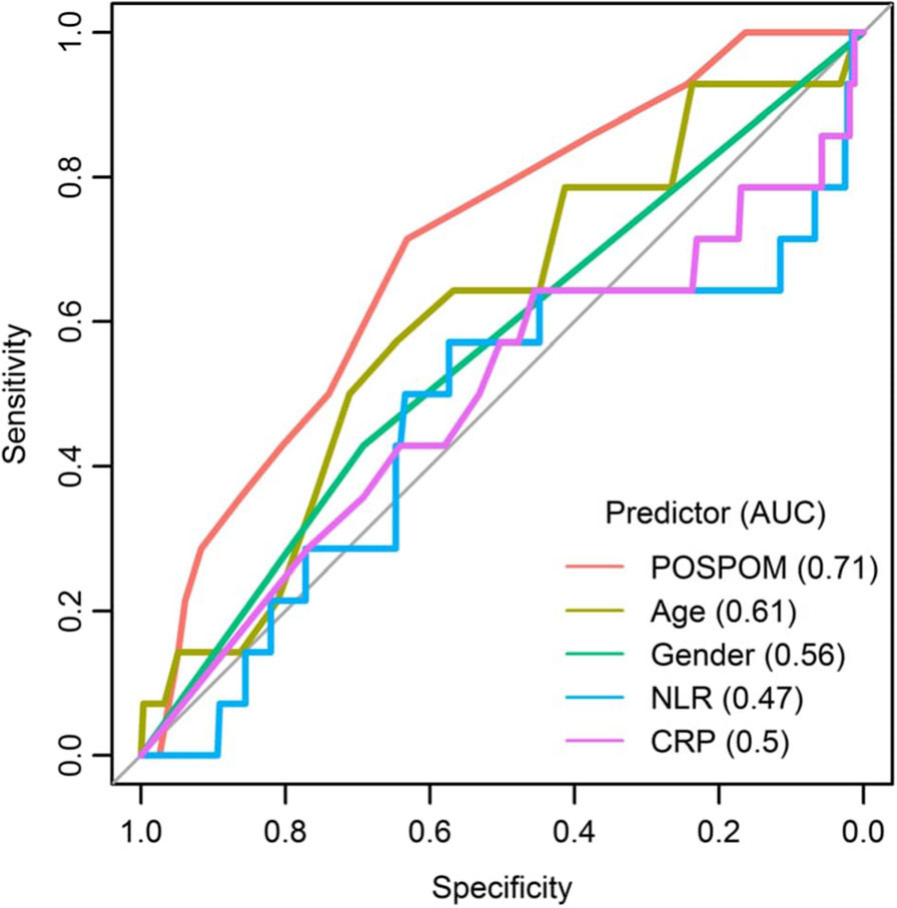
ROC curves of the restricted dataset and of each variable (univariate linear regression model)

Subsequently, age, male gender, NLR D_0_ or CRP D_0_ were combined to discriminate patients depending on their outcome. AUC for age, male gender, NLR and CRP were 0.608 [95% CI = 0.453–0.608], 0.560 [CI 95% = 0.424–0.560], 0.467 [95% CI = 0.285–0.467] and 0.498 [95% CI = 0.317–0.498] respectively (Fig. 3).

**Fig. 4:**
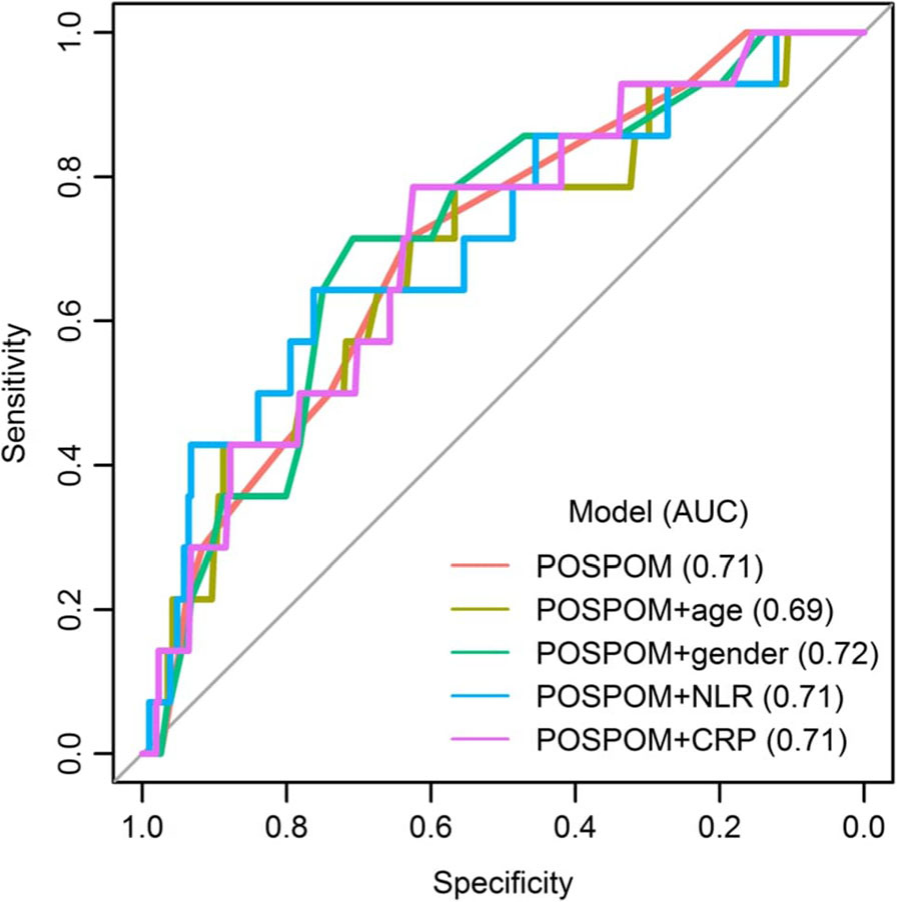
ROC curves of the combination of restricted dataset with each variable (bivariate linear regression model)

Finally, age, male gender, the NLR D_0_ and CRP D_0_ were individually combined with the POSPOM (Fig. 4). The predictive performance of the POSPOM is associated with an AUC of 0.705 [95% CI = 0.572–705] for mortality prediction. Each item was added separately to the POSPOM and the AUC was calculated. Neither age (AUC = 0.693; 95% CI = 0.544–0.693), gender (AUC = 0.717; 95% CI = 0.584–0.717), NLR D_0_ (AUC = 0.715; 95% CI = 0.563–0.715), nor the CRP D_0_ (AUC = 0.709; 95% CI = 0.573–0.709) improved the performance of the POSPOM.

### Other analyses

Despite the incapacity of the four investigated variables to predict postoperative mortality, the authors hypothesised that they may still remain as risk factors. A univariate linear regression model was therefore performed for each variable. The sole statistically significant result was the initial POSPOM setting, with a value of 0.17 ± 0.07 (*P = 0.019*). Results of the other univariate regression models for age, male gender, NLR D_0_ and CRP D_0_ were 0.053 ± 0.04 (*P = 0.20*), 0.52 ± 0.55 (*P = 0.34*), 0.05 ± 0.03 (*P = 0.12*) and 0.01 ± 0.015 (*P = 0.55*) respectively. **(**Table 3**).**

**Table 3:** Univariate linear regression model

Variable	Mean	Standard deviation	*P*-value
POSPOM	0.17	0.07	0.019
Age	0.053	0.04	0.20
Male NLR D_0_ CRP D_0_	0.52 0.05 0.01	0.55 0.03 0.015	0.34 0.12 0.55

Finally, a multivariate linear regression model including the POSPOM, age, male gender, NLR D_0_ and CRP D_0_ was created. The value for POSPOM was 0.15 ± 0.07 (*P = 0.046*), age 0.025 ± 0.04 (*P = 0.56*), male gender 0.25 ± 0.59 (*P = 0.67*), NLR D_0_ 0.058 ± 0.038 (*P = 0.12*) and CRP D_0_ 0.004 ± 0.016 (*P = 0.82*). Data revealed no significant result other than the validation of the discriminative capacity of the POSPOM. **(**Table 4**).**

**Table 4:** Multivariate linear regression model

Variable	Mean	Standard deviation	*P*-value
POSPOM	0.15	0.07	0.046
POSPOM + Age	0.025	0.04	0.56
POSPOM + Male POSPOM + NLR D_0_ POSPOM + CRP D_0_	0.25 0.058 0.004	0.59 0.038 0.016	0.67 0.12 0.82

## Discussion

The age, gender, NLR D_0_ and CRP D_0_ did not show discriminative capacity in predicting in-hospital mortality after HF. Furthermore, the addition of these variables to the POSPOM did not improve its performance.

Forget and colleagues previously identified age as a risk factor in elderly patients after surgery for HF [3]. Although age has already been integrated in the original POSPOM, we tested the variable independently in order to give it a higher importance in our scoring system.

The NLR as an inflammatory marker has proven its association with complications and long-term outcome in the setting of gastro-intestinal pathologies treated medically or surgically [6, 9–11]. The NLR has also been associated with complications after major cardiac events or in a variety of cancers [12, 13]. Forget and colleagues developed a four-item score which included the NLR D_5_ after surgery, to predict one-year mortality after surgery for HF [3]. Fisher and colleagues concluded in a recent paper that a high NLR at admission is an independent indicator of fracture risk in orthogeriatric patients and a significant risk factor and moderate predictor for intra-hospital mortality [7].

In our study, all CRP and NLR values taken at admission were included for analysis, simulating real life conditions. The score initially proposed by Forget and colleagues uses the NLR from a blood sample on D_5_, reflecting an inflammatory state [3]. The disadvantage of such a score integrating the NLR at D_5_ is the unavailability of the result on admission.

The calculated POSPOM score of our cohort predicts a mortality of 13.6%. This was higher than the 4.5% observed in our cohort. This difference may be explained by a multitude of causes.

First, our patients were followed-up using a multidisciplinary approach, the “co-care”-concept. This approach has been shown to reduce mortality compared with standard care [14]. In a meta-analysis and systematic review, Grigoryan and colleagues found that orthogeriatric collaboration was associated with a significant reduction in intra-hospital and long-term mortality, with a relative risk (RR) of 0.60 [95% CI = 0.43–0.84] and 0.83 [95% CI = 0.74–0.94] respectively. Hospital length of stay was reduced in the co-care model, with a standardised mean difference (SMD) of − 0.61 [95% CI = − 0.95, − 0.28] [15].

Second, in the validation paper published by Le Manach and colleagues, the percentage of major orthopaedic surgery (such as HF) was only 1.76% of total surgery [8]. Minor surgery was the most represented type of orthopaedic surgery, comprising 19.69%. Consequently, our study population of elderly HF patients was likely under-represented. Third, the optimal timing of surgery for HF in elderly patients still remains unclear [16–18]. Time to surgery was short in our study (81% of patients were operated within 24 h of admission). As detailed data of the POSPOM population was unavailable, we were unable to determine the significance of this variable.

Recently, several articles have been published showing a lack of accuracy of existing scores. For example, Boddaert and colleagues compared pre-operative surgical scores (ASA classification, POSPOM, Nottingham Hip Fracture score) and geriatric scores (Cumulative Illness rating Scale, Charlson comorbidity index) on a dataset of patients hospitalised in a peri-operative geriatric ward: They concluded no superiority in discriminative capacity of specific or geriatric scores in terms of short and long-term postoperative mortality prediction [2]. This result can be explained by the hypothesis that all of the scores are not designed to detect diminished physiological capacity in this frail population and suggest that more attention should be paid to frailty assessment rather than pre-operative characteristics, even in emergency conditions. A rapid, multidisciplinary clinical action plan in a shared ward for this patient sub-set, particularly designed to maintaining autonomy and reduce fall risk, could decrease postoperative mortality.

The intended heterogeneity in this cohort reflects a typical geriatric population and common clinical practice but can be considered as a potential source of bias. Furthermore, the relatively small number of events (32 deaths of 782 patients) limited the power of this study. With regards to the biological markers, the unavailability of the parameters in many patients limits the interpretation of these analyses. Specifically, no exclusion criteria were applied to obtained NLR values despite the fact that patients taking steroid therapy and smokers can show higher neutrophil counts. Patients with malignancies were also not excluded, thereby improving the generalisability of these results, whilst remaining a potential confounding factor. Furthermore, an ongoing infection is often a cause of confusion and fall in geriatric patients, resulting in high CRP D_0_ and NLR D_0_ values. Also, a malnourished state is frequently observed in elderly patients and is typically associated with lymphopaenia. Finally, the age-related impairment of the immune system may play a role in anomalies in this population [19].

## Conclusions

Age, gender, NLR D_0_ or CRP D_0_ have no discriminative capacity to solely predict postoperative mortality after surgery for HF. These variables do not improve the performance of the POSPOM, which remains poor in this population of geriatric patients scheduled for HF surgery.

## Abbreviations

AUC: Area Under the ROC-curve
CI: Confidence interval
CRP: C-reactive protein
D_0_: Day of admission
D_5_: Fifth day of admission
HF: Hip fracture
NLR: Neutrophil-to-lymphocyte ratio
POSPOM: PreOperative Score to predict PostOperative Mortality
ROC: Receiver Operating Characteristic
